# Retraction: Cucurbitacin E inhibits osteosarcoma cells proliferation and invasion through attenuation of PI3K/AKT/mTOR signalling pathway

**DOI:** 10.1042/BSR-2016-0165_RET

**Published:** 2024-02-28

**Keywords:** Akt, apoptosis, cell cycle, Cucurbitacin, mTOR signalling, osteosarcoma, PI3K, proliferation

This article is being retracted from *Bioscience Reports* at the request of the Editor-in-Chief and the Editorial Board following receipt of a notification from a reader, alerting the Editorial Board to images in this article that are published elsewhere including:

- Figure 2E B-actin, with Figure 4E B-action from Du et al 2017 (doi: 10.1186/s40659-017-0141-8)
- Figure 4A, with Figure 2F (Huh7) from Chang et al 2017 (doi: 10.1186/s12885-017-3216-6) and Figure 4B (HT29) from Sun et al 2016 (doi: 10.1042/bsr20160048).
- Figure 4F B-actin, with Figure 4A AKT from Gu et al 2017 (doi: 10.3390/ijms18040732)
- Individual tumour images from Figure 6F, that also appear in Tian & Yuan 2018 (doi: 10.1016/j.biopha.2018.06.070), Ruan et al 2019 (doi: 10.2147/cmar.s180418) and Zhang et al 2019 (doi: 10.1016/j.lfs.2019.116648)

The authors have been contacted with regards to the retraction and have not responded to the Journal's queries or the concerns raised. Given the extent of the issues raised, the Editorial Board stand by the decision to retract the article.

